# Deep bleeder acoustic coagulation (DBAC)—Part I: development and *in vitro* testing of a research prototype cuff system

**DOI:** 10.1186/s40349-015-0037-4

**Published:** 2015-09-18

**Authors:** K. Michael Sekins, Stephen R. Barnes, Liexiang Fan, Jerry D. Hopple, Stephen J. Hsu, John Kook, Chi-Yin Lee, Caroline Maleke, A R Ramachandran, Xiaozheng (Jenny) Zeng, Romain Moreau-Gobard, Alexis Ahiekpor-Dravi, Gareth Funka-Lea, Stuart B. Mitchell, Barbrina Dunmire, John C. Kucewicz, John Eaton, Keith Wong, Scott Keneman, Lawrence A. Crum

**Affiliations:** Siemens Ultrasound Business Unit, 22010 S.E. 51st Street, Issaquah, WA 98029-1271 USA; Siemens Corporate Research and Technology, 755 College Road East, Princeton, NJ 08540 USA; Center for Industrial and Medical Ultrasound, Applied Physics Laboratory, University of Washington, 1013 NE 40th Street, Seattle, WA 98105-6698 USA; ETN LLC, 1150 Guinda St., Palo Alto, CA 94301 USA; Medical Device and Technology Development and Commercialization (concultancy) , 8808 Points Dr. N.E, Yarrow Point, WA 98004 USA

**Keywords:** Ultrasound, Acoustic coagulation, Acoustic hemostasis, Acoustic thermometry, ARFI, Bleeding, Cautery, Combat bleeding, HIFU, Image compounding, Neural network, Recurrent neural network, Phantom

## Abstract

**Background:**

Bleeding from limb injuries is a leading cause of death on the battlefield, with deep wounds being least accessible. High-intensity focused ultrasound (HIFU) has been shown capable of coagulation of bleeding (cautery). This paper describes the development and refereed *in vitro* evaluation of an ultrasound (US) research prototype deep bleeder acoustic coagulation (DBAC) cuff system for evaluating the potential of DBAC in the battlefield. The device had to meet quantitative performance metrics on automated operation, therapeutic heating, bleeder detection, targeting accuracy, operational time limits, and cuff weight over a range of limb sizes and bleeder depths. These metrics drove innovative approaches in image segmentation, bleeder detection, therapy transducers, beam targeting, and dose monitoring. A companion (Part II) paper discusses the *in vivo* performance testing of an animal-specific DBAC system.

**Materials and methods:**

The cuff system employed 3D US imaging probes (“Ix”) for detection and localization (D&L) and targeting, with the bleeders being identified by automated spectral Doppler analysis of flow waveforms. Unique high-element-count therapeutic arrays (“Tx”) were developed, with the final cuff prototype having 21 Tx’s and 6 Ix’s. Spatial registration of Ix’s and Tx’s was done with a combination of image-registration, acoustic time-of-flight measurement, and tracking of the cuff shape via a fiber optic sensor. Acoustic radiation force impulse (ARFI) imaging or thermal strain imaging (TSI) at low-power doses were used to track the HIFU foci in closed-loop targeting. Recurrent neural network (RNN) acoustic thermometry guided closed-loop dosing. The cuff was tested on three phantom “limb” sizes: diameters = 25, 15, and 7.5 cm, with bleeder depths from 3.75 to 12.5 cm. “Integrated Phantoms” (IntP) were used for assessing D&L, closed-loop targeting, and closed-loop dosing. IntPs had surrogate arteries and bleeders, with blood-mimicking fluids moved by a pulsatile pump, and thermocouples (TCs) on the bleeders. Acoustic dosing was developed and tested using “HIFU Phantoms” having precisely located TCs, with end-of-dose target ∆*T* = 33–58 °C, and skin temperature ∆*T* ≤ 20 °C, being required.

**Results:**

Most DBAC cuff performance requirements were met, including cuff weight, power delivery, targeting accuracy, skin temperature limit, and autonomous operation. The automated D&L completed in 9 of 15 tests (65 %), detecting the smallest (0.6 mm) bleeders, but it had difficulty with the lowest flow (3 cm/sec) bleeders, and in localizing bleeders in the smallest (7.5 cm) phantoms. D&L did not complete within the 9-min limit (results ranged 10–21 min). Closed-loop targeting converged in 20 of 31 tests (71 %), and closed-loop dosing power shut-off at preset ∆*T*s was operational.

**Summary and conclusion:**

The main performance objectives of the prototype DBAC cuff were met, however the designs required a number of challenging new technology developments. The novel Tx arrays exhibited high power with significant beam steering and focusing flexibility, while their integrated electronics enabled the required compact, lightweight configurability and simplified driving controls and cable/connector architecture. The compounded 3D imaging, combined with sophisticated software algorithms, enabled automated D&L and initial targeting and closed-loop targeting feedback via TSI. The development of RNN acoustic thermometry made possible feedback-controlled dosing. The lightweight architecture required significant design and fabrication effort to meet mechanical functionalities. Although not all target specifications were met, future engineering solutions addressing these performance deficiencies are proposed. Lastly, the program required very complex limb test phantoms and, while very challenging to develop, they performed well.

## Background

Bleeding from vascular injuries in the arms and legs is the leading cause of preventable death on the modern battlefield. For the time in which this development program started, such wounds represented 50–70 % of all injuries treated (e.g., during Operation Iraqi Freedom [1]). While most injured soldiers can reach competent surgical and medical care within 30–90 min [2], due to tactical situations, many of the injured cannot reach such care until several hours after being wounded. The possibility of prolonged evacuation times, the need to prevent additional casualties, and the importance of completing the intended mission explain why casualties should be treated in the field.

In extremity vascular injuries in combat, bleeding occurs from superficial and deep vessels. Superficial wounds (e.g., <5 cm deep) are usually visually detectable and can be effectively treated with bandages, gauze sponges, or with recently developed hemostatic dressings, like HemCon® or QuickClot® [2].

D&L of deep vascular wounds, however, are much more challenging and are less accessible to coagulation intervention in the field. Further, their bleeding rates can vary widely, from severe hemorrhage (>500 mL/min) resulting in rapid irreversible shock to tiny occult bleeders (<5 mL/min), resulting in undetected fatal exsanguination over several hours. The project described herein^1 ^ was oriented to developing an ultrasound technology-based solution addressing the need to treat deep bleeding limb wounds in the field.

### Hemostasis: mechanisms of clotting (simplified)

In arterial injury, the hemostatic process, a complex “coagulation cascade,” starts when collagen in the vessel wall (e.g., in the adventia, the outermost layer) becomes exposed to blood. Platelets then adhere to the exposed collagen (the first stage of the cascade). During adhesion, platelets release clotting factors and become “activated” (second stage of clotting), recruiting and stimulating other platelets (among other functions), drastically changing the platelet shapes (e.g., spiny protuberances are formed), causing them to stick to each other (“aggregation,” the third stage of the cascade). Aggregation is assisted by fibrinogen, cross-linking the platelets to form a foundation for “plug” formation, in or along the vascular wall break. Aggregation, an active metabolic process, also involves fibrin to further cross-link the platelets, ultimately forming the insoluble fibrous network of the final hemostatic plug. Being a metabolic process, tissue heating thermally stimulates clotting by increasing platelet activation and the release of activating factors, enhancing aggregation. With sufficient heating, purely thermal mechanisms are upregulated and become significant contributors to coagulation.

**Fig. 1:**
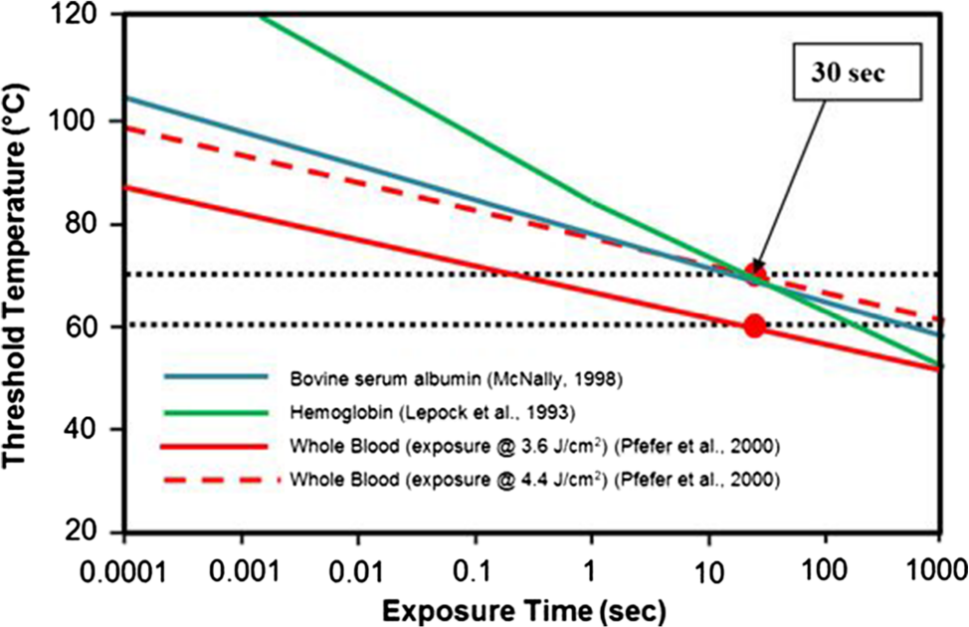
Coagulation threshold temperatures versus heating exposure time for blood and blood constituents (data taken from Pfefer et al., [4]). Other tissue coagulation criteria are included for comparison. For whole blood, the *H*_th_ = 4.4 J/cm^2^ curve was deemed the most valid experimentally. *Red dots* on the curves show 30 s (DBAC maximum dose time objective) exposure points and the corresponding coagulation temperature threshold target ranges (horizontal dotted lines)

Thermal coagulation of blood is therefore a rate process (Arrhenius equation behavior), rapidly accelerating above 60 °C *in vitro*, but appearing to have no further clotting changes above 80 °C [3]. For thermal coagulation to occur in 30–60 s, blood temperatures approaching 75 °C were required. Pfefer et al. [4], including data of other investigators for comparison, found a wide range of blood coagulation “threshold temperatures” (Fig. 1 shows their Arrhenius rate curves). For whole blood, to achieve coagulation with a 30-s exposure, the thresholds are approximately 60–70 °C.

### Acoustic hemostasis

In addition to its role in the coagulation cascade, movement of collagen into injury tracks or vessel wall breaks contributes to mechanical plug formation walls (note: collagen ≈ 1/3 the dry weight of arterial walls [5]). Vasculature contains type I and type III collagen, having fiber matrix structures capable of holding molecular water. In thermal acoustic hemostasis (acoustic cautery), collagen can be more dominant in plug formation compared to normothermic clotting. This is due to a combination of mechanisms, including heat’s ability to relax collagen and acoustic radiation force’s ability to “push” collagen and move cells (e.g., fibroblasts, collagen-secreting cells). Also with heating, water is driven out of vessel walls and collagen is denatured, cross-linking its fibers and shrinking it, stiffening and strengthening the adventitia in the vicinity.

HIFU is capable of producing hemostasis in vascular and organ puncture wounds [6–9]. In these studies, surface or superficial puncture wounds were treated with dose exposure times (*t*_dose_) typically greater than 1 min using hand-held HIFU devices. Arterial sealing involves blood coagulation combined with a “mechanical plug” involving native collagen, created from heating and tissue disruption (emulsification) [9]. As for other forms of cautery, acoustic hemostasis is also accompanied by localized acute tissue injury. Thermal doses associated with significant collagen shrinkage, measured as a function of tissue temperature history, are, in fact, above doses associated with cell death [10]. Importantly, in acoustic hemostasis of bleeding organs (liver and spleen) where tissues were heated >70 °C, complete healing and tissue regeneration within 60 days has been reported [9, 11].

### Acoustic regime for DBAC

Since the DBAC system is to be autonomously operated, treatment control and repeatability is paramount for safety. For this reason, the acoustic regime has been constrained to largely linear thermal mechanisms of coagulation, minimizing shockwaves, higher harmonics, cavitation, boiling, and non-linear behaviors associated with the high intensities needed for short *t*_dose_ lesioning. Avoiding gas production is desirable since bubbles are unstable and can either augment heating at the target or form a reflecting acoustic barrier, shielding power deposition [12]. Acoustic lesion formation associated with *t*_dose_ ≥ 10 s tends to be dominated by thermal mechanisms [13], and in the current project, *t*_dose_ ≤ 30 s was an objective. End-of-dose temperatures (*T*_eod_) at or above ≈70 °C have been shown to achieve thermal hemostasis in animal arterial puncture wounds using *t*_dose_ roughly on the order of 30 s [9, 10, 14]. Both collagen-centric hemostatic effects and blood coagulation have similar threshold temperatures in this *t*_dose_ regime (*T*_eod_ > 70 °C and *T*_eod_ ≈ 60–80 °C, respectively). Further, since 100 °C creates boiling, the dose objectives sought for DBAC were set at approximately 70 °C ≤ *T*_eod_ ≤ 95 °C. The DBAC treatment strategy is thus to create a cautery lesion (coagulation necrosis), with collagen shrinkage-associated fused reinforced “plugs.” Synergistically, supplemental bleeding reduction also comes from thermal constriction of small vessels.

### Cuff-based mechanical control of hemorrhage

Active bleeding during acoustic dosing can profoundly reduce coagulation since thermal energy and coagulation constituents are swept out of the region, and coaptation (apposition of vascular wall tissues) may also be compromised. Compensatory increases in acoustic power to overcome heat dissipation from active bleeding could be used but would increase the potential for superficial burns and push intensities toward non-linear regimes, affecting control and repeatability of dosing. For these reasons, the DBAC cuff design includes an integral tourniquet, operated to minimize, or completely stop, blood flow during acoustic dosing. The tourniquet alternates, automatically, between being constricted during dosing (inflated pneumatically or hydraulically) and being relaxed (deflated) during the bleeder targeting processes.

### DBAC cuff and program requirements

The work described herein was performed under United States Defense Advanced Research Projects Agency (DARPA) contract no. HR0011-08-3-0004. Milestone and target specifications referred to are those set between DARPA and the authors before the start of the project. A few minor modifications to these were allowed during the course of the project based on findings and insights gained. For example, the prototype cuff constructed needed to be only 3/4 of the length of the specified full (80 cm long) cuff.

**Fig. 2:**
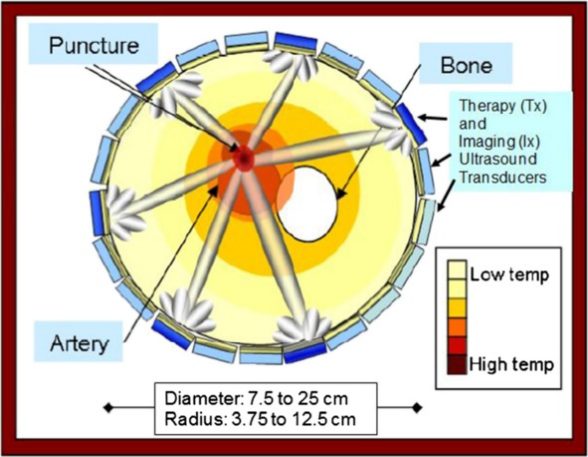
DBAC cuff concept (cross-section). Cuff on a limb with deep bleeding arterial punctures. Coagulation approach enabled by bleeder imaging from multiple 3D imaging probes (Ix) and HIFU treatment from multiple therapeutic arrays (Tx)

The DBAC device uses ultrasound imaging arrays (Ix) to detect and localize the arterial bleeding sites at depth and then uses HIFU arrays (“tiles” or Tx) to cauterize them. The intended product would be a lightweight, portable, and highly automated DBAC “cuff” (Fig. 2), which would be rapidly installed on the injured limb by a fellow soldier. Inherent in the cuff approach is the advantage of simultaneous confocal therapeutic beams delivered from the circumference of the limb, improving (over single HIFU-transducer treatment) the concentration and localization of heat at the target, while providing a larger aperture at the skin to reduce cutaneous heating. Although the program required that DBAC be tested *in vivo* (see [15]), most core performance functions had to be evaluated quantitatively, which required controlled *in vitro* test phantoms.

The prototype cuff device and its performance were subject to the following specifications: the cuff had to weigh ≤4.8 kg and address bleeders in limbs ranging in diameter from 7.5 (small arm) to 25 cm (large thigh), corresponding to minimum and maximum radius of curvature requirements (MinRC = 3.75 cm; MaxRC = 12.5 cm,). Related to these, bleeder depths (depth of penetration) had to range from 3.75 (MinDP; small arm) to 12.5 cm (MaxDP; larger limbs). The cuff had to be flexible and configurable to adapt to the different limb sizes. The dose (therapeutic focused power deposition) requirements at the bleeder site were defined by a minimum thermal dose (MinTD), i.e., 70 °C < *T*_eod_ < 95 °C (*in vivo* equivalent). For phantom testing, this translated to 33 °C ≤ ∆*T*_eod_ ≤ 58 °C, i.e., using equivalent temperature elevation (∆*T*_eod_) based on 37 °C body temperature^2^. Further, the dosing had to achieve MinTD at the target over a minimum therapeutic volume (MTV) (here, an 8-mm spherical region, reflecting the need to coagulate an adequate puncture wound volume^3^). These thermal criteria had to be met without exceeding a maximum thermal skin dose (MTSD)^4 ^= *T*_eod_ ≤ 52 °C, or ∆*T*skin_eod_ ≤ 20 °C. To minimize the risk of energy deposition outside of the target area, a maximum thermal tissue dose (MTTD) specification required that no significant heating occur outside of a 1-cm radius region (lateral to beam axis) around the target. Regarding targeting, D&L requirements included resolving minimum arterial (and puncture) diameters (minimum structure resolution, MSR = 0.6 mm) and slow bleeder flow velocities (minimum detectable velocity, MDV = 3 cm/s). D&L time was limited to 5 min for the first bleeder and 2 min for subsequent bleeders, with all MinTD therapeutic doses administered within 30 s. Metrics on automated operation permitted only two commands, one to start D&L (targeting) and one to start therapy, with targeting and treatment required to be closed-loop feedback processes.

## Materials and methods

### Cuff configuration and acoustic architecture

**Fig. 3:**
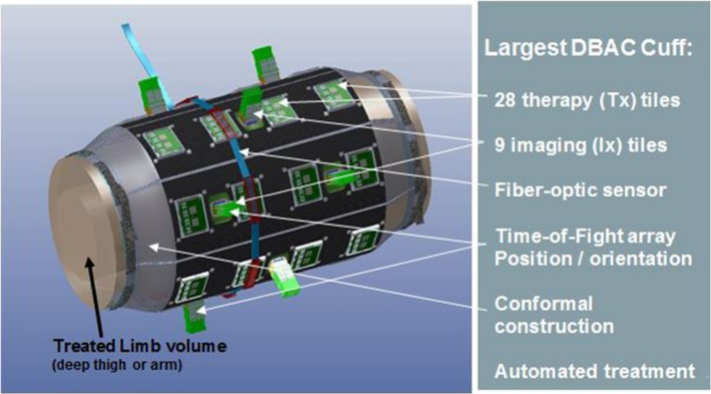
DBAC “full cuff.” Specified largest limb (*L* = 80 cm, *D* = 25 cm) cuff architecture, with therapeutic tiles (Tx’s) and 4Z1C imaging probes (Ix’s)

A commercial imager, the Siemens ACUSON SC2000™ ultrasound system (http://www.healthcare.siemens.com/ultrasound/cardiovascular/acusonsc2000-ultrasound-system) with multiple Siemens 4Z1c matrix array volume (3D) imaging probes (*f*_c_ = 2.5 MHz) (http://www.healthcare.siemens.com/ultrasound/ultrasoundtransducer-catalog; see 4Z1c probe category) provided the imaging core functionality for D&L, targeting, and acoustic thermometry. Cuff weight and imaging and therapy coverage dictated the optimal number and placement of ultrasound arrays; the specified full cuff design was 80 cm in length and included 9 Ix’s and 28 Tx’s, placed on seven cuff “panels” (Fig. 3).

**Fig. 4:**
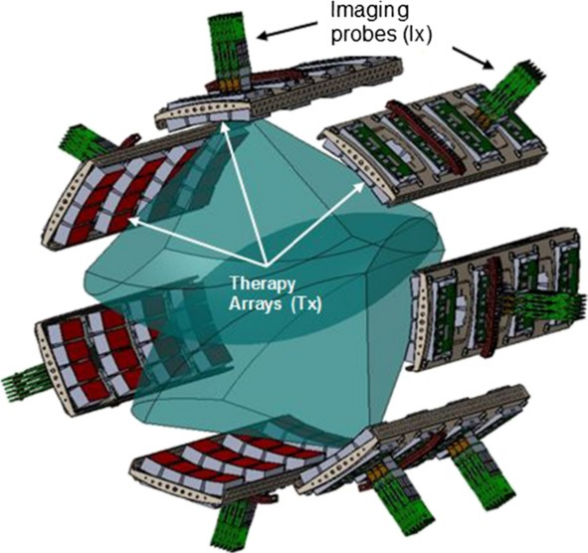
DBAC Cuff architecture, with discrete “panels” holding Ix and Tx arrays. Overlapping imaging sectors (blue pyramidal 3D volumes) enabled D&L coverage of deep arterial bleeders (5–12.5 cm deep on larger limbs). The seven-paneled cuff was suited to the largest limb (25-cm diameter thigh)

Imaging coverage was simulated in cuff-on-limb CAD models using 3D sector pyramidal image volumes characteristic of the 4Z1c (Fig. 4). Similarly, therapeutic coverage models used the focal depths and beam steering capabilities of the Tx’s.

#### Cuff prototype

The cuff device tested was permitted to be a “3/4 cuff” (60 cm in length) due to test phantom fixture and phantom fabrication limitations. Accordingly, the proof of concept cuff comprised 6 Ix’s and 21 Tx’s. Two ACUSON SC2000 systems were used in the prototype system, since each supported three Ix’s, and thus avoided the development of an image probe multiplexing switch. The DBAC software (SW) allowed imaging modes to be sequenced within the treatment steps and workflow.

### Therapeutic approach

The DBAC treatment is based on depositing acoustic energy (“doses”) at or near the bleeder site(s) in timed HIFU exposures of *t*_dose_ seconds. Adequate total acoustic power (*P*_dose_ [Watts], the superposition of that delivered from each selected Tx) must be delivered accounting for tissue attenuation, steering losses, perfusion energy dissipation, and focal power scanning patterns appropriate to the MTV size. In a DBAC product, after a dose is complete and cool-down achieved, bleeder status would be evaluated by the D&L subsystem to determine the need for subsequent doses.

### Cuff automation and user interface

The DBAC operational commands were limited to two user interface “buttons:” (1) Start D&L and (2) Start Therapy. The first button command initiated the detection and localization sequence, and at the conclusion of D&L the bleeding site therapy target markers were shown on the 3D vascular tree image on the system display, which also included the centerlines of the vessels. In addition, a listing of the number of vascular bifurcations and bleeders found was provided for operator inspection before the bleeder coordinates were transferred to the therapy subsystem.

Since D&L was to localize the first bleeder in ≤5 min, with subsequent bleeders in ≤2 min (e.g., ≤9 min for three bleeders), the Start D&L button launched a system timer to record these sub-process durations. The Start Therapy command launched the sequence to load the bleeder coordinates in the Tx frame of reference, select appropriate tiles, perform closed-loop correction of the beam foci to the target location, and deliver the therapy dose.

### Automated delivery of the thermal dose

**Fig. 5:**
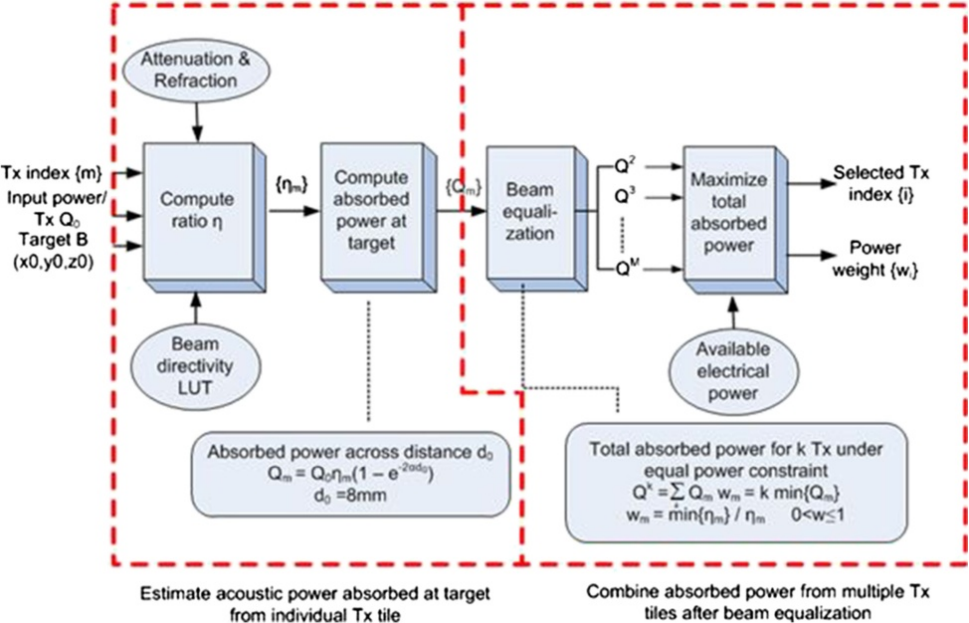
Automated Tx tile selection and dosing algorithm

The total Tx power requirements were estimated by analyzing doses based on absorbed acoustic power in 8 mm diameter MTV volumes, within the ranges of accessible bleeder depths and locations. A “power equalization” algorithm was used to balance, at the target, the absorbed power from each Tx, which also helped preserve the desired shape of the heated region, in that more Tx beams (subject to equalization) led to more spherical therapeutic volumes. As illustrated in Fig. 5, the therapy control algorithm accepted the target location and the list of “available” Tx’s, where available refers to those within distance and steering range of the target with unobstructed acoustic paths. The optimal combination of tiles (based on their acoustic relationship to the target, including the location of bones in beam paths) was automatically determined and the weighted absorbed powers of the selected tiles defined. The output powers for each candidate tile, calculated based on the steering angle (using a beam directivity lookup table [LUT]) of each focused beam and the tissue path to the target, were calculated.

The absorbed power within a spherical tissue volume of diameter *d*_0_ around the focal plane was estimated by

1 $$ {\mathrm{Q}}_{\mathbf{m}} = {\mathbf{Q}}_{\mathbf{0}}{\boldsymbol{\upeta}}_{\mathbf{m}}\left(\mathbf{1},, \hbox{--},, {\mathbf{e}}^{-\mathbf{2}\boldsymbol{\upalpha } {\mathbf{d}}_{\mathbf{0}}}\right), $$

where *Q*_*m*_ is the absorbed acoustic power produced by the *m*th Tx, *Q*_0_ is the maximum power capacity per Tx, *η*_*m*_ is the transport efficiency (ratio of acoustic power arriving at the target to input electrical power) for the *m*th Tx, and *α* was the attenuation coefficient of the tissue. A ranking method was then employed to determine the optimal number of tiles. The Tx’s were ranked by *η*_*m*_, in descending order. The first *k* tiles were grouped together, where *k* = minTx, minTx + 1, …, maxTx, where the minimum and maximum number of tiles, minTx and maxTx, were pre-specified. For each tile group, the powers from the *k* beams were equalized, and the total powers from all groups compared. The Tx group with the maximum total power was selected. The recruited Tx list was then provided to the algorithm, along with the power weighting factors, *w* (0 < *w* ≤ 1).

### Therapeutic arrays

**Fig. 6:**
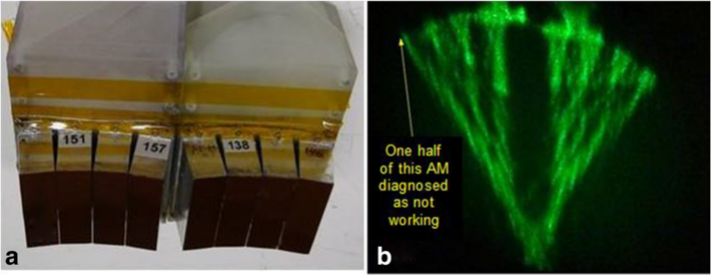
Therapeutic tile test fixture and Schlieren beam testing. **a** Test fixture holding two tiles (Tx) and **b** Schlieren image from the combined Tx beams, here confocal to same target location. Each tile comprised four acoustic modules (AM); image shown in (**b**) detects a transmission fault in one half of the AM on the far left

Compact arrays were developed employing a 52 × 13 mm aperture Acoustic Module (AM; 96 × 12 elements, azimuth and elevation, respectively) as the basic working unit for the therapy tile. After testing each 1152 element AM, four were assembled into a tile (Fig. 6a). The Tx was lightweight (≈115 g), with the beamforming electronics integrated into the array using ASICs-on-flex, and each AM required only a simple serial shift register-based interface (3 wires) and a power connection. A connector/buffer printed circuit board (PCB) allowed mounting of flex circuit data connectors and power connectors to the Tx module. Each 4-AM tile (4608 elements) was beamformed as a single 2D array unit, and since the Tx had significant electronic beam steering (60° and 45° in azimuth and elevation, respectively) and rapid scanning, the need for array mechanical motion was avoided. Each tile had a programmable controller (“Tile Manager”) based on a digital signal processor, enabling each Tx to receive high-level commands such as target coordinates, dose time, interleave-timing with the imager, etc., via USB. The Tile Manager and control SW allowed flexibility and support of evolving system needs. As shown in Fig. 6b, Schlieren imaging assisted in verification of proper beamforming and was used to rapidly check the acoustic output from all AM sections. A high dielectric constant transducer ceramic was also used in the Tx’s, allowing high power at moderate voltages. By implementing high-efficiency acoustic design and appropriately tuning acoustic matching layers, acoustic powers of 170 W/tile were reliably produced, with peak powers of 200 W and a ratio of acoustic power out to the electrical DC power into the AMs exceeding 50 %.

### Cuff mechanical architecture

**Fig. 7:**
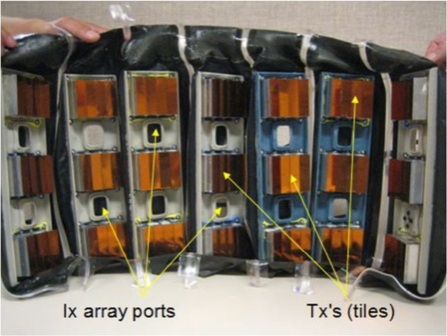
Fully Tx-populated seven-paneled cuff. Each panel had three therapy arrays (Tx’s) and one imaging array (Ix) installed. Not all 4Z1c imaging probe ports were simultaneously populated. This three fourth length configuration (21 Tx’s) embodied 96,768 therapeutic array elements

The prototype cuff had individual fabric panels, each with elastomeric end-sections providing sealing and conformability to the limb phantoms upon which they were tested, Fig. 7.

Bonded to the underside of each fabric panel was a lightweight rigid plastic frame which provided a mounting surface for the Tx’s and Ix’s. On each edge of the panel, a high strength, triple-bead Ziploc™-type zipper seal had been RF welded to the fabric. Since the fluid coupling compartment was filled with degassed water, the seals on the panel seams were used to provide a water-tight interface between each panel and allowed the cuff to adapt to the limb size; four panels for 7.5-cm limbs, and up to seven panels for the 25-cm limbs.

### Cuff system electronics architecture

The system electronics comprised: (a) a control PC (Dell workstation model T5400, Dell Inc., Round Rock, TX, USA) with custom SW enabling the DBAC operations and user interface; (b) three low-power analog and logic voltage supplies; (c) therapy power supply (3 kW, 150 V, 22 Amp variable supply, Agilent N8740A, Agilent Technologies, Santa Clara, CA, USA) powering all Tx’s (variable Tx voltages and duty cycle were used to control dose power); (d) two SC2000 ultrasound systems (enabling six 4Z1c transducers to be multiplexed); (e) interconnections, cabling, hubs, and instrumentation. A large custom 4-wire cable supplied power from the system cart to the cuff. Built into the wiring was a USB 2.0 hub for each panel, feeding into a single 8-port cuff USB hub. Data flowed from the imaging systems to the control PC, including imaging and thermocouple data (TC’s monitored via a DAQ USB device).

### Imaging modes and closed-loop control

DBAC system modes included D&L (B-mode, C-mode, power Doppler [P-mode] and spectral Doppler [SD] imaging modes), closed-loop targeting mode using thermal strain imaging (TSI), therapeutic power delivery monitoring via acoustic thermometry, and post-dose lesion assessment using B-, C-, and acoustic radiation force impulse (ARFI) stiffness imaging. The D&L architecture and imaging system control SW were modified to support automatic image acquisition, image registration, synchronous communication, time-critical algorithms, and a modified scan sequence for the Ix’s. The multiple overlapping volume images provided by the 4Z1c transducers on the cuff circumference (Fig. 4), after image stitching, gave 360° multi-angle, multi-view compounded Doppler flow images (“tomographic” C- and P-mode images). To meet DBAC fine structure imaging requirements (MSR) at deep locations and low flow sensitivity objectives (MDV), system level image SW changes had to be made. These principally involved increasing the Ix line density in the volume images. Closed-loop control required data communication pathways between modules in the DBAC system. The Image Analysis module processed the ultrasound raw (IQ) data, enabling both temperature estimation in therapy monitoring mode and beam focus localization in the targeting mode, with the resultant temperature map and the beam location plots rendered on the imaging system monitor display SW.

### Cuff software architecture

The DBAC SW platform (external PC) integrated several functions and controlled most operation of the prototype system, including Ix’s and Tx’s. The SW architecture was modular, offering flexibility, but with boundaries between functions. The system SW controlled: (a) external communication with the ultrasound imager, (b) image data receiving, (c) cuff control (including communication with the fiber optic subsystem, and time-of-flight (ToF) calibration (see below), (d) image stitching and compounding subsystem, (e) coordinate system management module, (f) reporting and visualization interface, (g) bleeding D&L module (analyzing compounded power Doppler image volumes to find bifurcations and automatically placing SD gates on each bifurcation), (h) the therapy subsystem (instructing delivery of power from multiple Tx’s to a particular coordinate in tissue space), and (i) therapy guidance (including automatic tile selection, dosimetry calculations and prescription, and closed-loop iterative targeting focal corrections). The SW system was able to sequence between all of the above functions almost completely automatically.

### D&L subsystem

The D&L process was made up of registration and image formation, detection and localization algorithm execution, and bleeding characterization. An image-based registration algorithm was used, providing precise knowledge of the relative positions of each Ix’s image volume (needed for the volumetric compounding in the 360° view of the whole limb). The multiple Ix views also improved detection of specular reflectors, removed speckle noise in B-mode images, and filled in images of vessel segments (C- and P-mode) that would be otherwise missing due to Doppler-angle dropout. Automatic vessel segmentation was performed and a visual model of the shape and size of the vascular tree was displayed by auto-mapping the vessel centerlines, after which the bifurcations were determined as the intersection of centerlines. Once a bifurcation was identified, three SD gates were automatically placed at the distal, proximal, and in-the-bleeder locations, at the prescribed distance from the bifurcation point. To determine whether a branch was a bleeder or not, the arterial resistive index (RI = [*V*_systole_ − *V*_diastole_]/*V*_systole_) was computed on each SD waveform for each outgoing branch. RI is affected by vascular resistance and vascular compliance; the “venting” of flow also contributes to altering compliance. Noting the RI for a bleed is lower than for normal flow, a threshold RI < 0.75 was used to define a bleeder.

### Cuff, array, and tissue registration

The known numbers of cuff panels determined the cross-sectional cuff geometrical shapes available (square, pentagon, hexagon, and heptagon). These were used, with image registration to the tissue, to define a calibration file that provided an initial estimate on transducer positions and orientations for D&L. Two methods for accelerating and fine-tuning array positions and orientations were used, that of fiber optic tracking and time-of-flight measurement.

Local curvature of the flexible conformal cuff and angles between panels (and thus transducer orientations) could be estimated in real time via fiber optic sensors (Shape-Tape™, Measurand Inc., Fredericton NB, Canada) wrapped on the cuff circumference. This reduced the search space for the image-based registration and, hence, its computation time, while increasing robustness of the overall image volume stitching. This being established, it was found that the flexible prototype cuff had enough rigidity to render fiber optic tracking optional. Thus, to reduce cuff weight, the Shape-Tape device was not deployed in the *in vitro* test bed final exam.

**Fig. 8:**
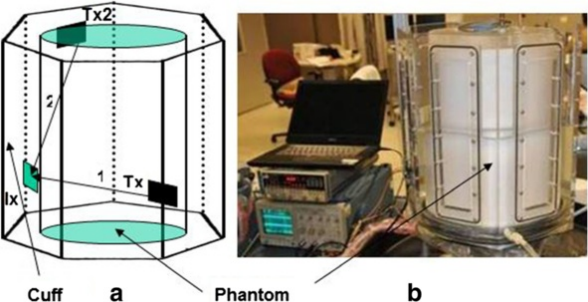
Time-of-flight array position and orientation determination. Distances between sub-apertures on Tx and Ix arrays were used (see the left panel a) for ToF array and cuff geometry determination (panel a). Photo (**b**) shows the seven-sided cuff water tank, mimicking a ToF process in the largest DBAC cuff

Using beamforming techniques and sound transmissions between pairs of sub-apertures on both the Ix and Tx arrays, the distances between the sub-apertures were determined via ToF, along with the speeds of sound in the phantom tissue and water layers (see Fig. 8). ToF calibration experiments utilized a seven-sided, rigid, acrylic water tank mimicking the largest limb cuff (Fig. 8b) and provided for mounting Tx’s and Ix’s in a cuff configuration. The method estimated locations of 3 × 3 mm sub-apertures on Tx tiles and then, using triangulation, determined the positions and orientations of all of the Ix’s and Tx’s. The results were compared to registration images (calibration file-based registration), and maximal registration errors were quantified.

### Closed-loop targeting

**Fig. 9:**
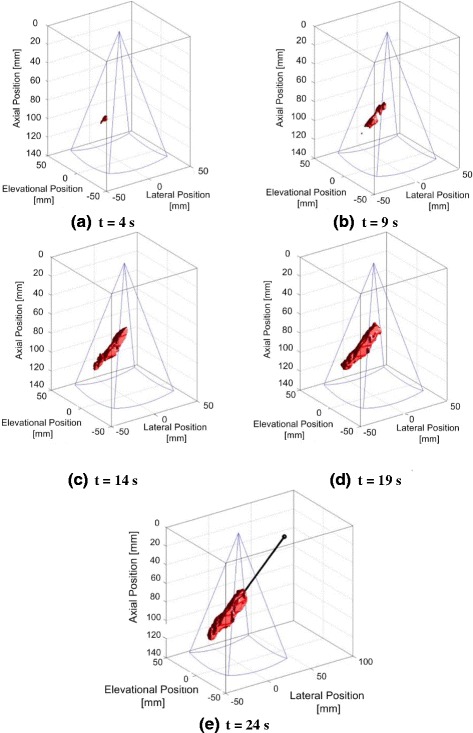
Visualization of a targeting beam focus based on segmented thermal strain imaging (TSI). Here, an extended low-dose exposure is used and visualized at (**a** through **e**): *t* = 4, 9, 14, 19, and 24 s. The Ix is located at position [0, 0, 0] mm and the imaging volume outlined by the *blue-lined pyramid*. The final, algorithm-determined approximation of the targeting beam (*black line*) and the therapy tile aperture centroid (*black circle*) are shown in (**e**)

After bleeder localization and the matrix of recruited Tx’s had been determined, the target locations were transformed into the local coordinates of each Tx. Because a flexible cuff and real tissue result in imperfect panel positioning and alignment, in speed of sound errors, phase aberrations, and focusing errors, misalignment between the detected bleed location and true initial focus of each Tx is expected. Accordingly, feedback-controlled iterative correction of Tx beam foci was implemented. This proceeded by each recruited Tx first sending a short, low-power therapy array test pulse to the D&L-determined tissue target coordinate. Sequencing through all recruited Tx’s, the image location of the focus and the beam-axis center line for each Tx was defined by either ARFI or TSI and an automated algorithm. The algorithm segmented the beam radiation pressure-induced tissue displacement pattern (volume) from the stationary tissue background, fitted a straight line to all data in the segmented volume, and searched for the maximum strain (i.e., peak temperature rise) location, as illustrated in Fig. 9a–e. For each figure, the plotted data is in the Ix coordinate space, and the Tx is on the top right, with a 45° angle beam propagating down to the lower left. The images show a progressively growing volume of increased thermal strain over 24 s. In real time, the system display plotted the beam axis as a line and the peak strain point (focus) as a blue dot. This “line and dot” approach provided visual feedback on the difference vector between the intended and actual focus locations for therapy, as an automated beam correction algorithm iterated until the actual focus overlapped the true target location within a specified targeting tolerance distance (<3 mm).

### Closed-loop dosing

A variety of acoustic methods were explored to monitor the DBAC HIFU dose in real time. Theoretical analyses, literature review, and experimental test comparisons were made for alternative methods, including ARFI, harmonic motion imaging, thermal echo-strain imaging, B-mode imaging of lesion formation, transient cavitation, and others. Because the DBAC system was to achieve coagulation through thermal mechanisms and because bio-effects have been validated using thermal dose relationships based on tissue temperature history (e.g., Sapareto [16] and Dewey [17]), expressed in cumulative equivalent minutes at 43 °C, the use of tissue temperature was preferred for DBAC dose monitoring.

To monitor tissue temperature, a recurrent neural network (RNN) thermometry method was developed [18–20] based on training algorithms to recognize temperature-associated changes in echo signals received from the heated regions where temperatures were recorded by thermocouples (TC). By combining multiple features of the echo signals (e.g., apparent tissue displacement due to radiation force, thermal strain, echo backscatter intensity changes, echo signal cross-correlation coefficients, etc.), weighting these in time and analyzing them in a neural network, estimated temperature changes at the treated region could be retrieved in real time and more accurately than TSI thermometry [20] alone.

### ***In vitro*** test bed for DBAC cuff evaluation

A two-phantom approach was adopted for the *in vitro* testing using (1) an “Integrated Phantom” (IntP) for assessing D&L and automated closed-loop targeting and dosing, including accuracy and speed, and (2) a thermal dose limb phantom (“HIFU Phantom”) to assess adequacy of HIFU power deposition while adhering to the maximum skin temperature requirement.

### Acoustic power deposition measurement

**Fig. 10:**
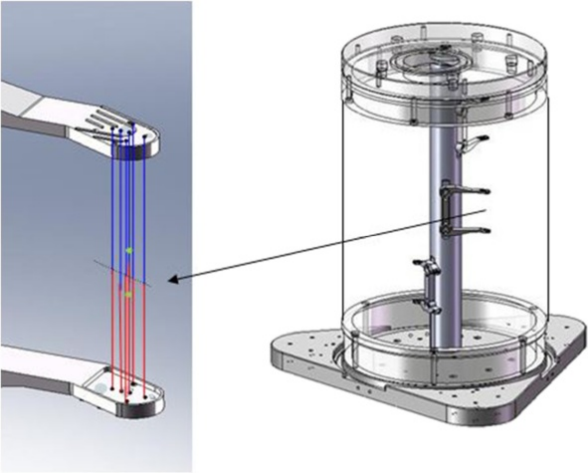
Therapeutic power HIFU phantom. Used to assess adequacy of the acoustic temperature elevation (Δ*T*) in TMM from the cuff multi-array simultaneous dosing. A thermocouple cluster (shown on the *left*, with 6 TC’s/cluster), sampled an 8 mm diameter spherical volume (MinTV). Each 60-cm long phantom had three TC clusters at specific coordinates for three vertical locations (*lower*, *upper*, *middle*) in the phantom

The therapy (HIFU) phantom was formulated from Gelrite™ [21] tissue-mimicking material (TMM) and had thermocouple (TC) clusters at specified locations representing bleeder targets, including prescribed depths (Fig. 10). The MinTD objective was measured by the thermocouple junctions at the TC clusters when focused power was delivered to the targets by multiple confocal Tx beams.

The risk of energy deposition outside of the target area, the MTTD criterion, was evaluated via simulations and hydrophone acoustic measurements. Also concerning safety, the MTSD skin temperature resulting from a maximal dose was recorded by TC measurements on the surface of a phantom. A target depth of 7.5 cm in a 15-cm diameter phantom was dosed with 55-W acoustic/tile, the maximum power/tile used in the *in vitro* test bed.

### Bleeder detection and closed-loop targeting evaluation

The DBAC closed-loop treatment process, evaluated in IntP tests, included: (a) detection of the bleeder by the D&L subsystem (including characterizing its blood flow waveform to classify it as a bleeder or non-bleeder); (b) localizing the bleeder coordinates via the D&L subsystem; (c) communicating these coordinates to the therapy subsystem, (d) automatic selection of the most suitable Tx arrays, (e) finalizing targeting by iterative adjustment of the Tx foci location (using either ARFI or TSI localization feedback); (f) treating the bleeder using the recruited Tx’s; (g) monitoring doses with acoustic thermometry; and (h) controlling therapeutic power with thermometry feedback; i.e., automatically shutting off power upon tissue temperature reaching a pre-set threshold value. The IntP was also used to evaluate the MSR, MDV, MinDP, and MaxDP and to check and confirm that phantom size (MinRC and MaxRC) requirements were met.

**Fig. 11:**
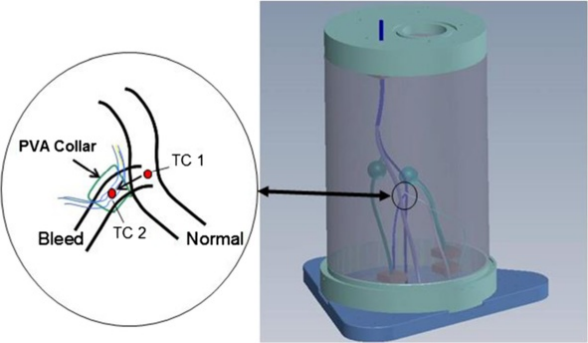
A 25-cm Integrated Phantom (IntP; on the *right*) and thermocouple arrangement on bleeder branch (*left inset*). The vascular tree was constructed of PVA; the soft tissue TMM (agar/gelatin/Al_2_O_3_/propanol) was poured and cast around the vasculature. Spherical vascular cavities were used to mimic hematomas

The Integrated Phantom was made from an agar/gelatin mixture, incorporating aluminum oxide particle scatterers and propanol concentration adjustments to set the speed of sound property [22]. The IntP not only had soft tissue acoustic and thermal properties but also possessed representative mechanical tissue properties (elasticity). Appropriate elasticity was required since targeting was accomplished using HIFU beam focus localization based on either ARFI imaging or (in the test bed) TSI principles, and because the acoustic thermometry methods were also dependent on tissue mechanical properties. One limitation in the IntP testing was that its TMM formulation could not withstand high temperatures (*T*_eod_ associated with full power thermal dosing), so adjustments had to be made on the dosing requirements. Both the IntP and HIFU phantoms were tested in each of three limb diameters: 25 cm, 15 cm and 7.5 cm. As indicated in Fig. 11, each IntP incorporated a polyvinyl alcohol (PVA) vascular tree with an undisclosed number of both “normal” vascular bifurcations and “bleeders.” Bleeders were PVA (10 %) vascular branches [23] leading to flow ducts (tracts) that were vented to atmosphere.

**Fig. 12:**
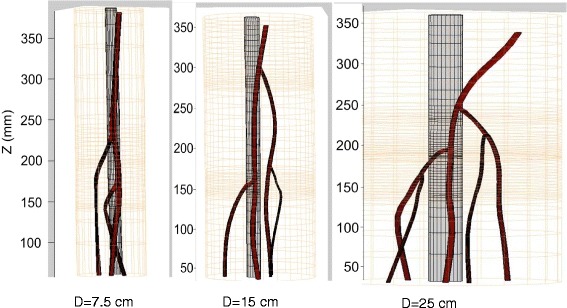
Representative Integrated Phantom vasculature geometries derived from HRCT scan images. Each limb size shown with embedded “vasculature” and “bone” surrogates

A blood-mimicking fluid (BMF) [24] was circulated in the IntP vascular system with pressure and flow waveforms delivered by a cardiac simulation pump (Model 551838 Pulsatile Blood Pump, Harvard Apparatus, Holliston MA, USA). At least two different flow configurations were used for each IntP guided by three SD flow velocity waveform images for each vessel/bleeder branch location (waveforms at pre-branch [upstream], branch 1, and branch 2). Thermocouples (0.075 mm, Type T) were placed along the longitudinal axis of the bleeder branches, oriented to face the skin surface, with the TC junctions 20 mm downstream from the point of bifurcation with main vessel. The TC was attached to the PVA vessel using ethyl cyanoacrylate adhesive. A single metallic bead was also glued to each TC 1 cm downstream of the TC junction as a fiducial marker for imaging. Two TC’s were used at each branch location, to compensate for potential rotation of the vessels during phantom fabrication. By standardizing the position of the TC’s on the PVA vessels, no targeting coordinate information was required. The vascular structure was imaged by high-resolution x-ray computed tomography (HRCT) prior to testing (Fig. 12), which enabled the verification of the presence and location of the TC beads. B-mode imaging and image registration was evaluated with the assistance of cross-shaped fiducial structures embedded in the IntP, and SD and P- and C-modes required a BMF for blood flow imaging.

## Results

### Cuff dosing simulations

**Fig. 13:**
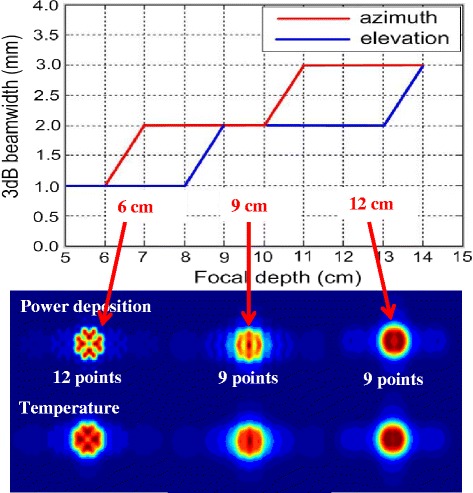
Simulated dithering patterns. Power deposition and temperature heating patterns with 3-dB focal beam dimensions, at three depths

Acoustic, thermal, and fluid dynamic computational simulations, combined with mechanical CAD models, were used to guide the therapeutic acoustic dosing of the cuff. A therapeutic frequency *f*_c_ ≈ 1 MHz was selected, optimizing between depth of penetration and acoustic absorption for heating. The Tx’s foci lateral dimensions (3 dB beam widths) were ≈1.0–3.7 mm (f# = 0.75–2.5), for small to large phantoms, respectively. By executing rapidly scanned dithering patterns, tailored heating patterns and laterally enlarged therapeutic volumes could be achieved. Focal dithering patterns were explored and optimized through acoustic and thermal simulations and confirmed experimentally. Separate dithering patterns were selected for three different DBAC depth ranges: (a) ≤ 7 cm, (b) 7–10 cm, and (c) ≥10 cm (Fig. 13). Rapid movement of the focus (switching rate up to 10 kHz) between 12 dithering points were chosen for focal depths ≤7 cm, and 9 points were used at all other depths.

**Fig. 14:**
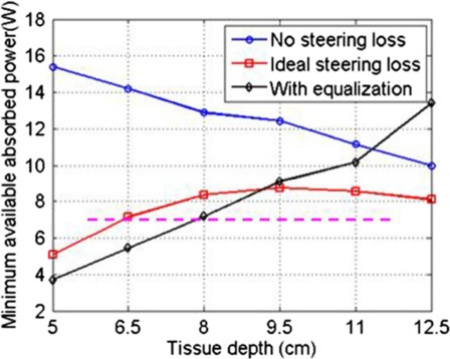
Estimated minimum available absorbed acoustic power as a function of depth. *Blue curve* assumes power loss from attenuation only (3 Tx’s, no equalization); *red curve* has attenuation and beam steering losses (3 Tx’s, no equalization). The *black curve* assumes attenuation and steering losses (6 Tx’s, beam equalization applied)

The total Tx power requirements were estimated based on absorbed acoustic power occurring within 8 mm diameter MTV volumes. Based on simulation, ≈4 W of absorbed power at the target was found to achieve the minimum ∆*T*_eod_ = 33 °C after a 30-s dose. The lowest available (absorbed) power for targets at different depths in a 25-cm limb (worst case power requirement) is plotted in Fig. 14. All curves were above this absorbed power threshold at all depths. Experimentally, 3.5 W absorbed was found sufficient for ∆*T*_eod_ = 33 °C in the Gelrite TMM phantoms. Intensity at the skin was limited to ≤6 W/cm^2^ to avoid skin burns (based on skin burn energy thresholds of LaCoste et al. [25], scaled to the utilized *t*_dose_ values). The maximum power allowed from a single Tx was thus determined to be 163-W acoustic.

### ***In vitro*** test bed summary

The results of the in vitro performance evaluation are shown in Table 1. The time consumed to install (wrap) the cuff around the limb phantoms, including adding or removing panels to adapt the cuff to limb sizes, improved over the course of the testing: from ≈1.5 h to <30 min. The cuff met weight limits, being 4.75 kg in the monitored weigh-in of the final exam.

**Table 1:** Summary of the *in vitro* test bed objectives and results

Program milestone	Phantom type			
Objective	Integrated Phantom (D&L/targeting)	Result	HIFU phantom (power delivery)	Result
Minimum structure resolution (MSR)	PVA vessel 0.6 mm diameter	Met goal (2/2)	NA	NA
Minimum detectable velocity (MDV)	PVA vessel 3 cm/s mean velocity	Did not meet goal (0/3)	NA	NA
Min and max thermal dose (MTD)	NA	NA	Non-feedback test 6 thermocouples (Δ*T*: 33 °C ≤ TD ≤ 58 °C)	Met goal
Min and max depth of penetration (MDP)	Min: 5 cm	Met goal (1/2) Met goal (3/3)	Min: 5 cm	Met goal
	Max: 12.5 cm (25-cm diameter phantom)		Max: 12.5 cm (25-cm diameter phantom)	Met goal
Maximum thermal skin dose (MTSD)	NA	NA	Surface thermocouples (MTSD: Δ*T*_max_ ≤ 20 °C)	Met goal
D&L on minimum radius of curvature (MRC) phantom	3.75-cm depth bleeder in a 3.75-cm radius phantom	Did not meet goal (0/5)	3.75-cm depth in a 3.75-cm radius phantom	Met goal
D&L algorithm	Madsen w/PVA vessels	Met goal	NA	NA
D&L to targeting communication	Two thermocouples on vessel wall to verify targeting	Met goal	NA	NA
Targeting to therapy power communication and coordinated functioning	Closed-loop targeting and dosing w/o human interaction	Met goal	NA	NA
Closed-loop targeting	“Line and dot” measured	Met goal	NA	NA
Closed-loop dosing	RNN thermometry	Met goal	NA	NA

### Power delivery

**Fig. 15:**
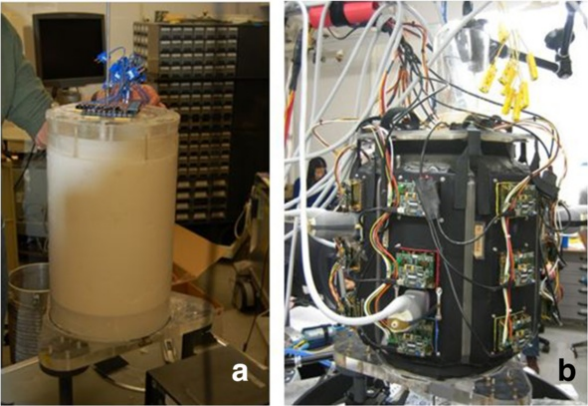
DBAC cuff therapeutic power assessment using a HIFU phantom. **a** A 25-cm Gelrite phantom and (**b**) seven-paneled fully populated cuff mounted on the phantom for MinTD (power delivery) assessments

**Fig. 16:**
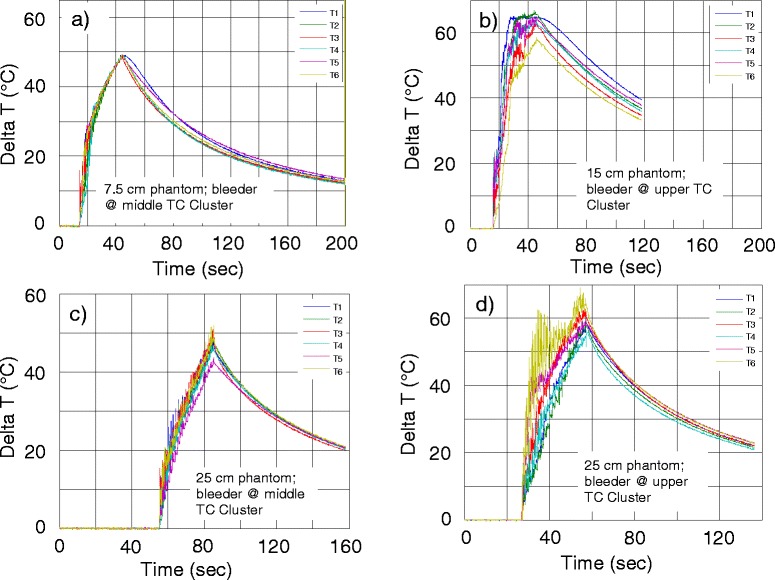
Sample thermal responses from multi-beam dosing in Test Bed HIFU phantoms. Results from the small (Panel a), medium (Panel b) and large (Panels c and d) diameter phantoms are shown. Sample thermocouple Δ*T* temperature histories measured with DBAC cuffs on different-sized phantoms at specific thermocouple clusters

A seven-paneled DBAC cuff is shown in Fig. 15 installed on a 25-cm diameter HIFU phantom. A representative set of thermal responses to 30-s power doses for each phantom size is shown in Fig. 16. The thermal dose criteria (MinTD, ∆*T*_eod_ = 33–58 °C) were substantially met (Table 2), with minor qualifications (see Table 2 footnotes), including one bleeder site that had an excessive ∆*T*_eod_ (curve T6 in Fig. 16d). Assessing the risk of energy deposition outside of the target area, the MTTD analysis indicated the strongest side lobe to be −9 dB below the main-lobe, producing no significant heating outside the 1 cm radius around the target. The measured skin temperature resulting from a maximal dose was well below the MTSD, with measured skin ∆*T*_eod_ ≈ 0.6 °C.

**Table 2:** Summary of *in vitro* therapeutic power (MinTD) results

Phantom	Cluster position	Cluster depth (cm)	Number of Tx tiles	HIFU focus (offset relative to center of cluster) (mm)			Input acoustic power (W)	Focal intensity (W/cm^2^)^a^	∆*T* (°C)
				*X*	*Y*	*Z*			
7.5 cm	Lower	3.75	2	−0.08	0.26	0.11	57.2	316	45–47
7.5 cm	Middle	3.75	2	0.04	0.01	−0.02	56.4	316	49
15 cm	Lower	5.5	3	0.55	−0.32	−0.83	98.2	316	39–55
15 cm	Middle	4.5	3	0.21	−0.06	−0.05	83.8	316	48–51
15 cm	Upper	7.5	2	NA	NA	NA	93.8	316	57–65^b^
15 cm	Lower (B)	7.5	2	NA	NA	NA	83.9	316	35–42
25 cm	Lower	12.5	3	0.12	−0.33	−0.3	167.0	316	43–49
25 cm	Middle	8.0	3	−1.7	1.2	0.48	168.4	316	16–45^c^
25 cm	Middle (B)	8.0	3	−0.74	−0.44	−0.02	144.7	316	42–48
25 cm	Upper	5.0	3	−0.61	−0.04	1.0	134.0	316	55–62^d^

*NA* coordinates not available due to data recording error

^a^Intensity value spatially averaged over focal dithering pattern cross-section for each Tx beam focus then derated for attenuation along each beam path for beams; Intensities for all foci then superposed

^b^Atypical curve shape with enhanced echogenicity observed in the TMM around the cluster

^c^Coordinate value entered incorrectly

^d^Strong oscillation present in TC readings; possibly indicating viscous heating artifact (see Fig. 16d above)

### D&L

**Fig. 17:**
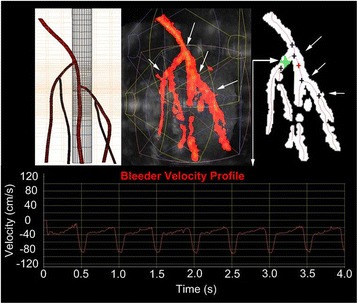
D&L images in 25-cm Integrated Phantom. (*Upper left*) Model derived from high-resolution CT scan showing the 4 branches, (*center*) power Doppler compounded volume. The *white arrows* indicate branches that are detected (*right*) automatically by the algorithm. The spectral Doppler (SD) waveform (*lower*) shows a bleed detected at the location of the *green marker*. Resistive index (RI) = 0.70

Detection and localization was successful overall but with better localization found in the larger phantoms (15 and 25 cm), as shown in Table 3. Branch detection (see Fig. 17) was evaluated by the ability to identify the actual vessel branches in the phantom (extra branches were ignored unless a false bleeder). SD acquisition was scored by the ability to obtain a blood velocity waveform for every branch identified. Bleeder characterization required the correct identification (bleeder versus normal).

**Table 3:** Summary of D&L success rates

Sequence	7.5 cm	15 cm	25 cm
B/P-mode acquisition	8/8	2/3	4/6
Branch detection	7/8	3/3	2/5
Spectral Doppler (SD) acquisition	8/8	2/2	5/5
Bleed characterization	7/8	2/2	4/5
Tile recruitment	6/6	2/2	5/5
Target correction	4/6	2/2	4/5
Dose sequence	5/6	2/2	4/4
Dose feedback	5/5	2/2	3/4
Localization	0/5	2/2	1/4
Automation	4/7	2/3	3/5

Localization refers to the bleeder being accurately targeted and correctly dosed. The automation requirement specified that the complete 2-button sequence proceed without unexpected human interaction; i.e., the B- and P-mode acquisitions, tile recruitment, target correction, dose sequence, and dose feedback were all scored based upon completing the sequence without problems (e.g., no SW lockups). The 2-button sequence was demonstrated with only minor pre-declared “manual” steps being used (that being manual transfer of imaging probes from one ACUSON SC2000 system to the other). Sixty percent of the test runs were completed using full automation, and no system crashes occurred. Fully automated successful D&L and treatment of bleeders occurred for single fast bleeders in the 25- and 15-cm IntPs. This included a deep bleeder (11.8-cm depth) in a 25-cm IntP, a shallow bleeder (5.4 cm deep) in a 25-cm IntP, and a minimum structural resolution bleeder (MSR = 0.06 cm) in a 15-cm IntP. Successful targeting of dual bleeders in the 25-cm phantom was demonstrated (both at ≈5-cm depths), but one of these targets was dosed beyond the set IntP temperature limits. A triple bleeder in a 15-cm IntP was also successfully completed (including the MSR bleeder). At least two full-automation sequences were completed in the 7.5-cm IntP, but no proper localizations were achieved in this sized phantom.

As shown in Table 4, D&L did not meet time limit requirements for three bleeders (i.e., <9 min), largely due to increased image acquisition times. The total acquisition time for the 7.5-cm phantom was 5 min 40 s for power Doppler image acquisition, with an additional 4 min for characterizing all bleeds (total = 9 min 40 s). Similarly, D&L time for the 15-cm phantom was 11 min, and for the 25-cm phantom was between 18 and 21 min.

**Table 4:** Acquisition times (s) during *in vitro* final exam

Phantom	Number of panels	Power Doppler (average)	Bleeding characterization (average)	Total time (s) (maximum)
7.5 cm	4	340	240	580
15 cm	5	425	240	665
25 cm	7	1020	240	1260

### Acoustic thermometry

**Fig. 18:**
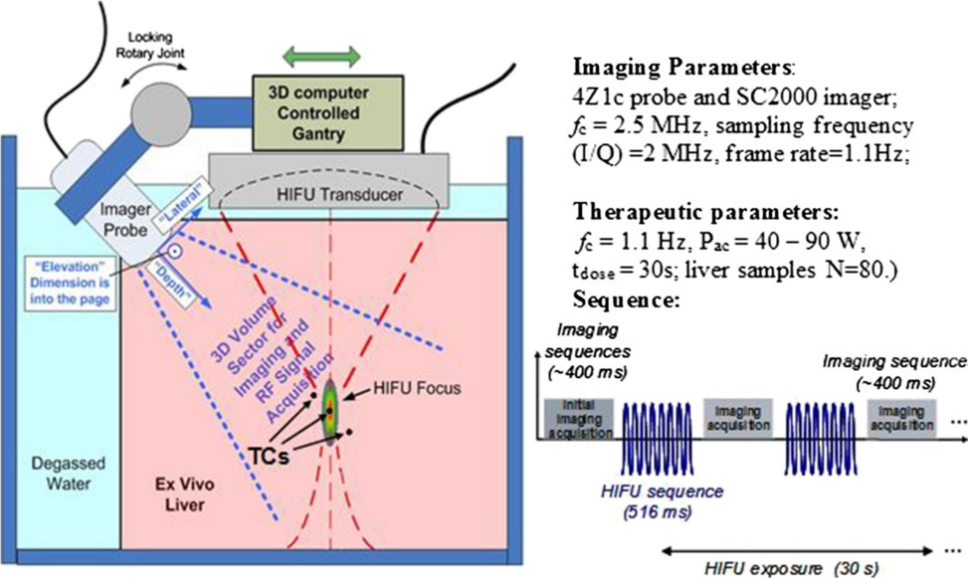
Acoustic thermometry experimental setup. Test fixture configuration for acquiring ultrasound echo data during HIFU heating experiments for thermometry data acquisition and RNN testing. Imaging and therapy interleave in sequence shown

Thermometry experiments were performed for RNN algorithm training purposes in both TMM and *ex vivo* livers (Fig. 18). A total of 251 datasets at different HIFU power levels were obtained from IntP TMM (54) and *ex vivo* bovine livers (197) and then processed offline. Needle TCs or butt-welded TCs were inserted into the tissue media to read and output the target temperatures, which were acquired using a TC DAC (Model USB 2416; Measurement Computing Corp., Norton MA, USA) at 54 Hz throughout the heating process.

**Fig. 19:**
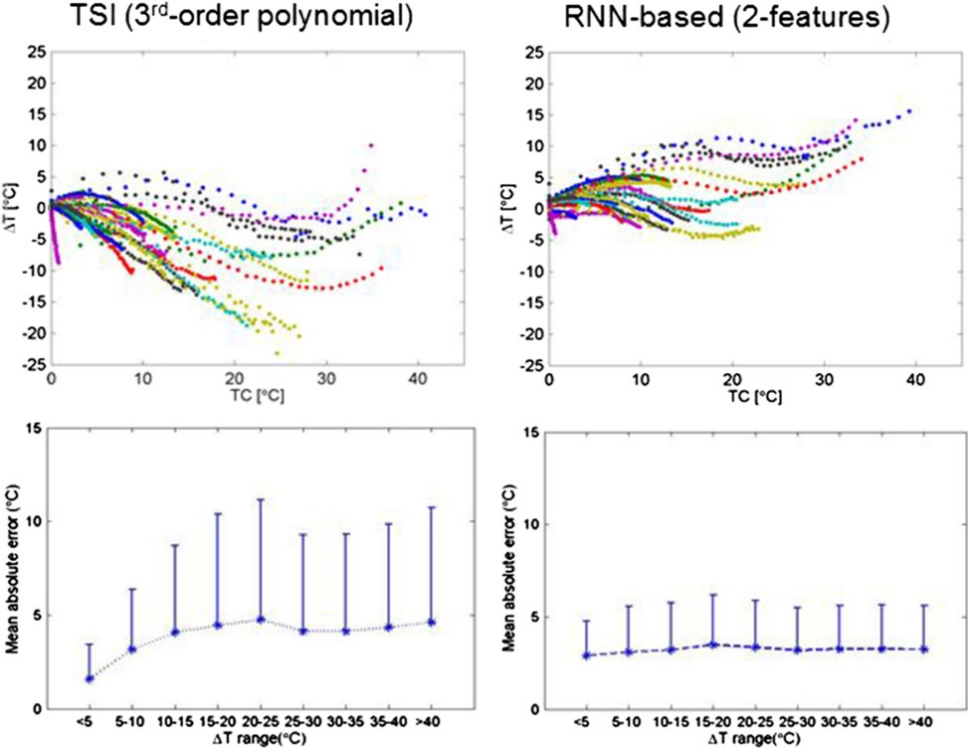
RNN versus thermal strain acoustic thermometry. Mean absolute error of one RNN method versus TSI—using third-order polynomial fitting). TSI average strain = −0.23 ± 0.05 % to −0.81 ± 0.06 % for ∆*T* = 2.3 ± 1.1 to 7.71 ± 3.2 °C, with a calculated linear coefficient (*λ*) = 0.0012 °C^−1^. Thus, for ∆*T* > 10 °C, TSI underestimated Δ*T* by ≈33 %. RNN thermometry average errors *E* ≈ 2.5 °C for ∆*T* = 0 to 10 °C and *E* ≈ 3.8 °C for ∆*T* = 11 to 30 °C

Figure 19 shows representative performance of the RNN algorithm [20], here compared to TSI as has been used by others [26]. As indicated, the RNN approach appears superior to TSI in this application, particularly in the higher ∆T’s needed for a therapeutic range, and also distributes error more equally across the entire range of temperatures.

### Closed-loop targeting

**Fig. 20:**
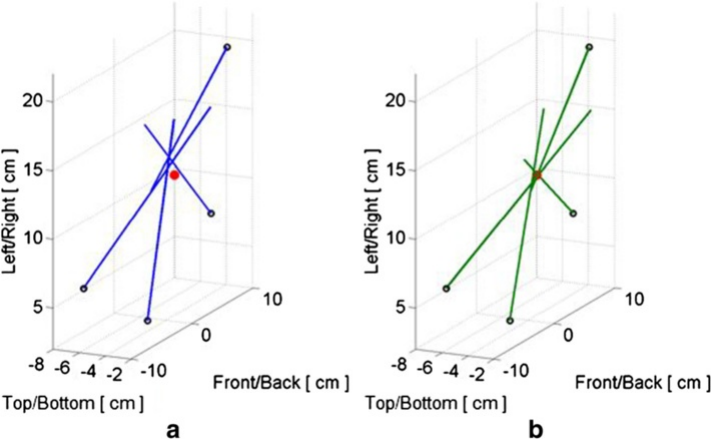
Targeted beams before and after closed-loop correction. Detected targeting beam axes from four therapy tiles (seven-sided cuff on 25-cm IntP), whose aperture centroids are marked (*black dots*). **a** The initial D&L-determined axes and **b** axes after a single targeting-correction iteration (each converging laterally to well within the 3-mm targeting tolerance)

Closed-loop targeting was scored on the ability of the targeting algorithm to iteratively converge the beam focus from each recruited Tx to a location superimposed on the detected bleeder location within a tolerance of 3 mm. The utility of the algorithm is illustrated in Fig. 20 for the 25-cm IntP and the seven-paneled cuff. After selecting the target location on a bleeder track bifurcation, thermal dosing was performed twice with four Tx’s; once using the D&L calculated foci (Fig. 20a), and then done after closed-loop targeting correction (Fig. 20b). As shown, none of the initial foci were sufficiently close to the desired target location, whereas after a single iterative correction, closed-loop targeting converged for all four tiles to the desired target location.

**Fig. 21:**
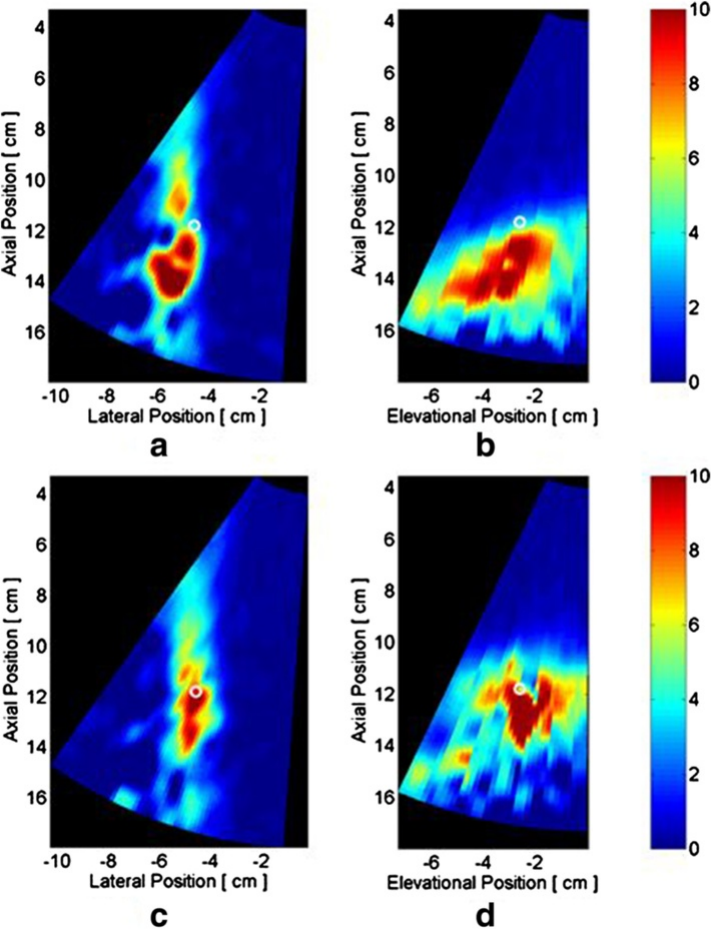
Temperature rise (°C) contour maps due to 30-s dosing under targeting conditions of Fig. 20. (**a**) and (**b**) are D&L location-targeted beams, while (**c**) and (**d**) show heating after iterative correction of the beam foci. The desired target location is represented by the *white circle*

The resulting RNN temperature distributions after four-Tx dosing of 30 s, with and without closed-loop targeting, is shown in Fig. 21. Without closed-loop targeting (Fig. 21a, b), the temperature images indicate heating occurred across a large volume, particularly in elevation, and deeper than the target location. After closed-loop targeting correction convergence (Fig. 21c, d), the temperature rise is better concentrated within a smaller volume that also encompasses the target location.

**Fig. 22:**
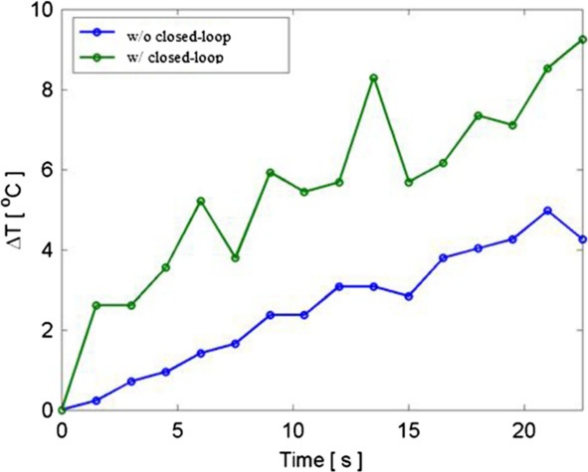
Thermal response before and after targeting corrections. Temperature rise measured at the target location from a 22-s four-Tx dose. After closed-loop targeting (*green curve*), the temperature rise at the desired focus is greater than without closed-loop targeting (*blue curve*)

Figure 22 also demonstrates that without closed-loop targeting, convergence mistargeting would result in less concentrated energy delivery, decreasing the heating of the bleeders.

Of 65 targetings, 12 were focused within the convergence criterion after initial focusing (based only on D&L coordinates). Closed-loop feedback converged 31 of the remaining 53 foci to the target (58 %). It is noteworthy, however, that after increasing the iteration limit from three to five, the targeting convergence rate improved from 9 of 22 (41 %) to 22 of 31 (71 %).

### Closed-loop dosing

The IntP dosing control results are summarized in Table 5. Closed-loop RNN acoustic thermometry monitored the temperature, and when reaching the Δ*T* threshold, power shut-down was automatically triggered. The Δ*T* threshold was based on the RNN spatial average temperature within the MTV (8-mm region), and in all tests power shut-off was reached prior to the fixed exposure limit of 30 s. In general, the threshold-triggered power shut-offs were well behaved, with 60 % of the tests showing the peak TC end-of-dose temperatures close to the dose threshold set points.

**Table 5:** Summary of closed-loop targeting and feedback-controlled therapy

Phantom (by size)	Bleeder location	Number of tiles	Targeting convergence success rate	Dose time (s)	Δ*T* (eod): set point dosing threshold (temperature) (°C)	Δ*T* (eod): average RNN estimated temperature rise) (°C)	Δ*T* (eod): (TC measured temperature rise) (°C)
7.5 cm	Lower	3	2/3	11.2	5	5	1.5^a^
	Middle 2	2	2/2	8.3	5	5.8	n/a^b^
	Middle 3	2	0/2	8	5	5.1	2.2
15 cm	Middle	4	3/4	25.2	15	15.2	13.5^c^
	Upper	3	3/3	11.3	5	5.1	12/4^d^
	Lower	3	2/3	15.1	5	4.5	3
	Lower 2	2	2/2	13.2	5	6.8	6
25 cm	Middle	3	1/3	15	5	6	4.5
	Middle 2	3	2/3	12.6	5	5.7	2
	Middle 3	2	1/2	9.3	5	6	4
	Lower	2	2/2	18.75	5	5.5	30/10^e^

^a^Insufficient Δ*T*; thermal peaking cross-check revealed foci off the TC by *X* = 3 mm, *Y* = 5 mm, and *Z* = 6 mm

^b^Abnormal Δ*T* measured (decreasing); wiring check found no error. High-power dose shot used; damaged PVA vessel; dissection was needed to confirm the location of TC and condition of the vessel branch

^c^The first experiment; ∆*T* threshold = 15 °C and resulted in phantom vessel damage; subsequent ∆ thresholds set to 5 °C

^d^TC measured oscillatory temperature curves. Speculate multiple beams struck TC producing varying viscous heating artifact

^e^TC showed a smooth Δ*T* rise to 30 °C on one TC and an oscillatory Δ*T* response on the other TC

### Registration: ToF

In using the development test tank (Fig. 8), the locations of the sub-apertures estimated by ToF across water + TMM (Gelrite) were, encouragingly, quite close to the locations expected by the design specifications of the tank. The method also included aberration correction for refraction of the beam going through water layers into the tissue media. Ix–Tx distances across the Gelrite phantom, plus water jacket, were typically measured to within 2 mm of ground truth. The orientation of each Tx relative to an Ix could be measured to within 0.5° of the array normal surface vectors.

**Fig. 23:**
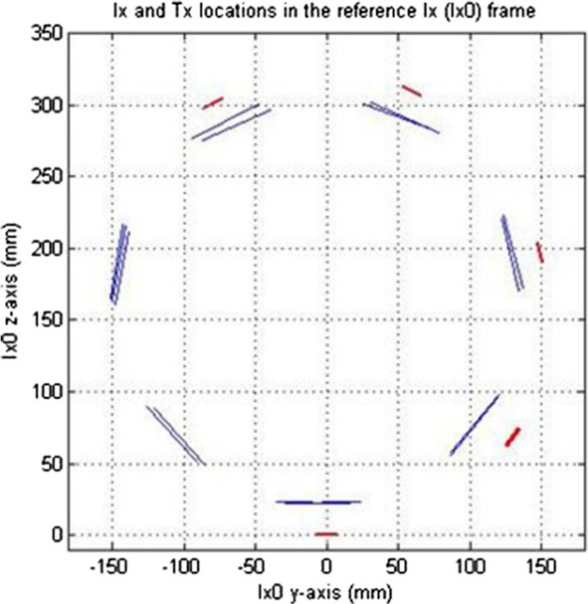
ToF imager and therapeutic array position and orientation projections. “Limb” cross-sectional view of Ix and Tx locations in Ix0’s coordinate system (in vitro test bed test, 25-cm Integrated Phantom); *blue lines* represent Tx’s and *red lines* represent Ix’s

In the final exam, ToF tests were only performed on the 25-cm IntP with the seven-paneled cuff. In one case, the ToF test was done with only 20 of the 21 Tx tiles and five of the six Ix’s being fully functional. In spite of this, the ToF subsystem correctly identified the cuff as having seven sides/panels, accurately estimated the acoustic velocity of the phantom TMM (at 1515 m/s) and the image registration comparisons showed a maximal registration error of less than 1 cm. The locations of the Ix’s and Tx’s in this case, projected onto the YZ plane in the reference imager (Ix_0_) frame, are shown in Fig. 23.

## Discussion

### Power delivery

Importantly, the thermal dose criteria (MinTD) were met in all tests, covering all three sizes of HIFU phantoms, with only minor qualifications. Due to the focal gain allowed by the use of multiple Tx’s treating a single tissue target, the measured peak skin temperature resulting from a maximal dose was far below the skin temperature limit. Further, the MTTD analysis showed no significant heating outside the 2-cm region around the target.

### D&L

In vitro results were positive and 1-button D&L automation workflow was verified in all tests, with a total of 65 % successful automatic localizations being shown in the final exam. Satisfying the MSR, two of three small (0.6 mm diameter) bifurcations were successfully identified and treated in the 15-cm phantom. While the first MSR branch was not detected successfully, this was likely because of the BMF scatterer dilution. The subsequent MSR bleeds were automatically detected after correcting the BMF concentration. As shown in Table 1, meeting the slow flow requirements (MDV = 3 cm/s) were challenging in that the Ix’s were not sensitive enough (via PW Doppler). Further, the resistive index criterion was less helpful with in vitro slow bleeds, as they often had irregular flow waveforms. In the future, these cases could be addressed by handling the slow bleed waveform as a special case, not using RI for bleeder characterization. Detections of flows 3–4 cm/s were achieved in the IntPs using a 2D imaging probe with more penetration (Siemens 6C2 transducer).

**Fig. 24:**
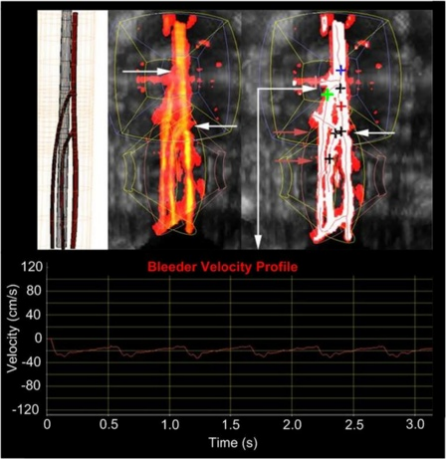
Small limb phantom bleeder branch detection. From 7.5-cm phantom test in the final exam: *upper left* is CT scan model, *center* is P-mode volume, showing two bifurcations, and *right figure* shows D&L results, here with two extra branches (*red arrows*). *Lower figure* is the spectral Doppler (SD) signal at the *green marker* (blood velocity waveform)

Although bleeder detection was possible in the small IntPs (7.5 cm), no successful localizations were scored in them. It is speculated that bifurcation localization was less precise due to the more narrow branching angles in the small limb phantom (see left panel in Figs. 12 and 24), noting that the algorithm had been trained on the larger IntPs. By determining the actual coordinates of the “bleeder” TCs via “thermal peaking” (where the Tx beam focus is auto-scanned until power pulses maximize the TC response), it was confirmed that systematic errors had occurred in the automated D&L-reported target coordinates. Also complicating the small limb D&L was the fact that the four-paneled cuff had imperfect Doppler coverage. The transducers that had the best image volume overlap were also the ones facing each other, so where one had a bad Doppler angle on a vessel, the other contralateral transducer likely had a similar Doppler blind spot. Solutions going forward include using more and smaller imaging transducers for the small limb cuff.

There were two cases where the Tx beam raised the temperature at a bleeder TC but not to the required minimum level. Positional checks of TC junctions via thermal peaking showed the beams to be within approximately 5 mm of the center of TC junction. A shift of 2–3 mm of the TC junction in the wrong direction could have put it just outside the 8-mm spherical range. Post study inspections indicated the TCs were nominally in the right locations, but because the junctions were not visible on x-ray, their exact locations could not be determined with high precision.

Regarding not meeting D&L time limits, for large phantoms and slow bleeders, the acquisition required an increase in the spatial resolution over that of the commercial 4Z1c. The imaging sequencing was, therefore, modified for these cases to use a higher line density. This required sequentially acquiring sub-volumes (fragments) of power Doppler image volumes (18 per probe) and then reconstructing the full volume from these sub-volumes in a post-processing step. This stretched the acquisition time budget to ≈18 min for a 25-cm phantom. For small phantoms, if “extra” bifurcation sites were detected (red arrows on upper right in Fig. 24), increased D&L processing also resulted (in this example, an increase of 120 s resulted). In the future, processing times can be improved by increasing the image-former memory, modifying image-former SW and small phantom D&L algorithm training.

### Closed-loop targeting

Closed-loop targeting was necessary for many treatments, particularly when multiple foci coming from the surrounding circumferential set of Tx apertures had to be coincident. It is encouraging that target localization was determined to be within 2.3 mm laterally of the true target and repeatable to within a beam angle of 1°. Closed-loop targeting and therapy automation were largely demonstrated in all IntPs. A few pre-agreed upon manual (“human in the loop”) steps were required, principally the manual switching of 4Z1c transducers between the two imaging systems after D&L but before the Start Therapy button was pushed.

### Closed-loop dosing

To our knowledge, the RNN acoustic thermometry method implemented was its first use in thermal therapy. The accuracy of the RNN temperature estimates, over a wide range of dose exposures in *ex vivo* bovine livers and TMM phantoms, was encouraging. The RNN-estimated temperatures largely coincided with or were close to the readings from TCs. From the 2D temperature maps, it was observed that the RNN algorithms were able to locate foci and beam axes of the Tx beams and were somewhat reliable when used to shut down power based on pre-set temperature threshold values.

The closed-loop dosing did exhibit some atypical behaviors deserving mention. For example, the proximity of vessels to the focal target region may have biased the spatial-average temperature estimates downward, likely due to acoustic distortions and/or cooler temperatures in the vessel lumen. In the most extreme case, the thermometry signal (averaged over the MTV volume) triggered power-off at Δ*T* = 5.5 °C (threshold set at Δ*T* = 5 °C), but the TC measured the vascular target at Δ*T* = 30 °C (Table 5, dose time = 18.75 s in 25-cm phantom). The TC signals had no obvious artifact and impedance tests and post-test dissection suggested the TCs were functioning properly. The RNN thermometry image in this case revealed high temperatures at the periphery of the target volume but a low-Δ*T* void in the central target area, likely the vessel lumen, supporting the notion of a bias due to temperature spatial averaging.

### Registration

Understanding and defining the spatial relationship of the therapy arrays to the tissue space, to each other, and to the imaging arrays are needed for targeting integrity. The registration solutions pursued (image-based, fiber optic Shape Tape, and time-of-flight methods) were compared in terms of accuracy, image region of interest, processing time benefit, and cuff weight limitations. D&L and image-based registration have no weight penalties, and image registration accuracy was good. ToF also had the advantage of no system weight and produced reasonable position and orientation information on Tx’s and Ix’s. Fiber optic cuff-shape tracking was used to accelerate image registration of the other two methods and did provide suitable initial estimates for cuff array position and orientation tracking, but its weight was a disadvantage. For this reason, the fiber optic hardware was not used in the test bed configuration of the cuff. ToF is most advantageous where highly variable cuff panel orientations are involved (as for real human limb treatments). The invariant geometries of the test bed cylindrical phantoms, however, were such that ToF registration acceleration benefit was not significant, so ToF was not integrated into the test bed DBAC system functionality. Successful ToF performance was demonstrated, however, laying the groundwork for its use in future cuff or similarly flexible HIFU systems.

### ***In vitro*** test bed facilities

Test specifications and DBAC device principles required the development of a variety of complex IntP and HIFU phantoms, along with their instrumentation and test protocols. The phantoms and test methodology worked well in the majority of cases in the final exam.

## Summary and conclusion

A research prototype cuff system was designed and fabricated to meet many of the challenging performance requirements of DBAC for battlefield trauma. The project had an accelerated development calendar which also limited the opportunity for multiple practice test sessions. The device was evaluated in a witnessed and refereed in vitro exam series. Requiring only minor trouble-shooting during testing, most DBAC cuff performance requirements were met, including cuff weight, power delivery (MinTD), targeting accuracy, skin temperature limit (MSTD), and autonomous operation (limited to two “button” commands; although a few manual steps were permitted). The cuff structure proved strong enough to support arrays, while being mechanically flexible and adaptable to the limb phantom size range, and also meeting the important cuff weight objective (≤4.8 kg).

Central to project success was the development and fabrication of compact, lightweight high element-density therapeutic arrays (Tx), capable of significant steering, rapid, precise focal scanning and high-power delivery. In light of the number of elements per array, the architecture allowed simplified electrical drive and control connections, a result of the electronic and transducer fabrication processes developed, including ASIC-on-flex design and construction. Also key was the availability of commercial wide-sector volume 3D imaging matrix array probes (Ix). The final cuff deployed 21 Tx’s and six Ix’s, for full coverage of the deep bleeder target tissue volume without the need for mechanical motion in the cuff.

“Two-button automation” (to launch D&L and therapy processes) was achieved, but in some tests required a few discrete manual steps since system SW integration had not been completed by the final exam date.

D&L, the first step in the DBAC treatment, integrated several functions: volumetric stitching to compound the images (overlapping P- and B-mode volumes) from all Ix’s in the cuff, and automatic image processing algorithms that enabled localization and characterization of the bleeders. D&L bleeder/non-bleeder classification was reliable via spectral Doppler measurement of resistive index, except in low flow bleeders. Registration was enabled using 3D image registration, fiber optic shape sensors, and ToF triangulation methods, complementary approaches with the potential to accelerate D&L image volume compounding.

D&L challenges included inferior bleeder localizations in the smallest diameter limbs, likely because of the narrow vascular branching angles in the 7.5-cm phantoms compared to the training phantoms. Also problematic was the detection of the slowest flow rate bleeders, in part due to Doppler sensitivity. The need to modify the imaging probe sequence, increasing the line density to improve 4Z1c imaging resolution, came at the cost of increased image acquisition times, exceeding D&L procedure time limits. In the future, re-optimization of the imager software for this application, supplemental algorithm training, plus adding more system memory should improve these results.

Closed-loop targeting was demonstrated, with targeting convergence for in vitro bleeders improving as the focus iteration algorithm was adjusted with experience, ultimately converging 20 of 31 targets. Closed-loop dosing was also operational, with control of therapy power shut-off triggered by pre-set IntP tissue ∆*T* thresholds. Key closed-loop technologies included RNN acoustic thermometry for dosing, thermal strain imaging of the Tx beam focus for targeting, and refined SW algorithms combining image processing with interactions between the Ix and Tx subsystems.

Although not detailed here, significant effort went into the development of the test phantoms (IntP and HIFU types) and the test protocols themselves, including generating multiple TMM formulations, doing material characterization studies, fabrication method improvement, and instrumentation development. Although some minor phantom variability occurred (e.g., fragility of TC’s and PVA vessel thermal sensitivity), overall, the tests were well executed and reliable.

In the in vitro device development effort, a majority of successful DBAC system milestones were delivered. After the in vitro test bed final exam, the project moved forward to the development and testing of DBAC in an animal model of limb bleeding (see companion Part II article [15]).

## Endnotes

^1^This research was, in part, funded by the US Government. The views and conclusions contained in this document are those of the authors and should not be interpreted as representing the official policies, either expressed or implied, of the US Government.

^2^The MinTD lower threshold (∆*T* ≥ 33 °C) was defined based on experience and literature indicating *T*_eod_ ~70 °C (33 °C above 37 °C core) were associated with hemostasis in dose exposures sought in this program (30–60 s). To maintain safe control under automated treatment, tissue boiling was to be avoided, thus a maximum of 95 °C was stipulated (i.e., ∆*T*_eod_ ≤ 58 °C from 37 °C core temperature).

^3^The therapeutic volume dimension for a discrete dose was that corresponding to the narrowest dimension across the coagulation lesion, *W*_min_, which exceeds the static acoustic focus waist dimension (e.g., FWHM of the effective beam), accounting for a margin due to thermal conduction. *W*_min_ minimum size was based on the size of the vessel treated and on targeting tolerance (error) margins. The largest artery encountered was the femoral artery (*D* ≈ 5 mm), and a targeting uncertainty of ±1.5 mm was determined, thus the therapeutic volume was set at 8 mm.

^4^A maximum *T*_eod_ = 52 °C was prescribed (i.e., ∆*T*_eod_ ≤ 20 °C from baseline skin temperature of 32 °C), approximately corresponding to skin burn threshold temperatures for the acoustic dose exposure time range used.

## Abbreviations

AM: acoustic module (working unit of the Tx tile; 4AM’s per Tx)
ARFI: acoustic radiation force impulse imaging
B-mode: ultrasound gray scale imaging
C-mode: color flow Doppler imaging mode
D&L: detection and localization; function/subsystem for finding bleeder sites
DAQ: digital signal/data acquisition device
DBAC: deep bleeder acoustic coagulation
HIFU: high-intensity focused ultrasound
IntP: integrated phantom; used for testing closed-loop targeting and dosing
Ix: imaging probe/transducer or imaging probe functions
MaxDP: maximum depth of penetration (deepest bleeder target) at 12.5 cm
MaxRC: maximum phantom radius of curvature treatable by the DBAC cuff; 12.5 cm
MDV: minimum detectable velocity: slowest detectable bleeder flow rate is 3 cm/s
MinDP: minimum depth of penetration (most shallow bleeder target); 3.75 cm
MinRC: minimum phantom radius of curvature treatable by the DBAC cuff; 3.75 cm
MinTD: minimum thermal dose: 70 °C < *T*_eod_ < 95 °C or 33 °C ≤ ∆*T*_eod_ ≤ 58 °C
MSR: minimum structural resolution: smallest detectable bleeder vessel is ≥ 0.6 mm
MTSD: maximum thermal skin dose: *T*_eod_ ≤ 52 °C, or ∆*T*skin_eod_ ≤ 20 °C
MTTD: maximum thermal tissue dose: no significant heating outside of a 1 cm radius (laterally to the beam axis) from target center location
MTV: minimum therapeutic volume (8-mm spherical tissue region at the target)
PC: personal computer; a desktop workstation computer
PCB: printed circuit board
P-mode: power Doppler flow imaging mode
RNN: recurrent neural network; acoustic thermometry method used for this project
SD: spectral Doppler
SW: Software
TC: Thermocouple
*T*_eod_: end-of-dose temperature
TMM: tissue-mimicking material used for soft tissue surrogates in phantoms
ToF: time-of-flight (method for determining Ix and Tx cuff positions and orientations)
TSI: thermal strain imaging
Tx: therapeutic array or therapeutic array functions
USB: Universal Serial Bus
Δ*T*_eod_: end-of-dose temperature rise relative to baseline temperature starting point
